# Enriching distinctive microbial communities from marine sediments via an electrochemical-sulfide-oxidizing process on carbon electrodes

**DOI:** 10.3389/fmicb.2015.00111

**Published:** 2015-02-17

**Authors:** Shiue-Lin Li, Kenneth H. Nealson

**Affiliations:** Department of Earth Science, University of Southern CaliforniaLos Angeles, CA, USA

**Keywords:** carbon electrodes, differential pulse voltammetry, electrochemical sulfide oxidation, marine sediments, microbial community analyses

## Abstract

Sulfide is a common product of marine anaerobic respiration, and a potent reactant biologically and geochemically. Here we demonstrate the impact on microbial communities with the removal of sulfide via electrochemical methods. The use of differential pulse voltammetry revealed that the oxidation of soluble sulfide was seen at +30 mV (vs. SHE) at all pH ranges tested (from pH = 4 to 8), while non-ionized sulfide, which dominated at pH = 4 was poorly oxidized via this process. Two mixed cultures (CAT and LA) were enriched from two different marine sediments (from Catalina Island, CAT; from the Port of Los Angeles, LA) in serum bottles using a seawater medium supplemented with lactate, sulfate, and yeast extract, to obtain abundant biomass. Both CAT and LA cultures were inoculated in electrochemical cells (using yeast-extract-free seawater medium as an electrolyte) equipped with carbon-felt electrodes. In both cases, when potentials of +630 or +130 mV (vs. SHE) were applied, currents were consistently higher at +630 then at +130 mV, indicating more sulfide being oxidized at the higher potential. In addition, higher organic-acid and sulfate conversion rates were found at +630 mV with CAT, while no significant differences were found with LA at different potentials. The results of microbial-community analyses revealed a decrease in diversity for both CAT and LA after electrochemical incubation. In addition, some bacteria (e.g., *Clostridium* and *Arcobacter*) not well-known to be capable of extracellular electron transfer, were found to be dominant in the electrochemical cells. Thus, even though the different mixed cultures have different tolerances for sulfide, electrochemical-sulfide removal can lead to major population changes.

## Introduction

It is important to understand the abilities of microbes to interact with insoluble electron donors and acceptors when investigating the microbial communities in any specific environment. For instance, dissimilatory metal reducing bacteria (DMRB), such as *Shewanella* or *Geobacter* are capable of extracellular electron transfer (EET) to solid substrates, such as Fe(III) (Myers and Myers, 1993; Bretschger et al., 2007), or Mn(IV) (Myers and Myers, 2001; Bretschger et al., 2007), Cr(VI) (Myers et al., 2000), V(V) (Myers et al., 2004), and U(VI) (Lovley et al., 1991; Gorby and Lovley, 1992). The addition of Fe or Mn oxides will thus enrich these groups, if they are present. DMRB can also reduce electrodes and have been applied in many microbial-fuel-cell (MFC) studies (Gregory et al., 2004).

It is logical that by applying the proper potential, an electrode could be used in place of a solid-state electron donor/acceptor for metal respiring bacteria, a fact that is now well-established (Kim et al., 1999; Bond et al., 2002; Okamoto et al., 2013). However, to the best of our knowledge, no study has conclusively found that a polarized electrode will restrict the growth of microorganisms that are not capable of generating current, as long as the major growth factors are sufficiently supplied. To this end, Torres et al. (2009) demonstrated that a biofilm on an electrode surface can form an insulating layer that would unexpectedly reject truly EET-capable bacteria like *Geobacter*. To obtain more specific results, it is suggested that the samples should be chosen from some distinctive environments, like those with high-ionic conductivity that may possess halotolerant microorganisms being competent in MFCs.

Marine sediments are complex high-conductivity, geochemically active ecosystems of major importance in the global carbon cycle, Madigan et al. (2012). These systems are characterized by a series of redox processes with oxygen, nitrate, manganese oxide, iron oxide, and sulfate reacting in that order (Nealson and Saffarini, 1994; Burdige, 2006). Because of the very high (≥25 mM) sulfate concentration, most marine sediments are dominated by sulfate reducing bacteria (SRB), and sulfide plays a crucial role in the oceanic environment (Burdige, 2006). Given these properties, and the knowledge that many bacteria are capable of EET, it is reasonable to utilize electrochemical approaches to study marine sediments: quantifying redox equivalents in the form of current. In addition, electrochemical removal of substrates like sulfide might also be used to reduce feedback inhibition, and facilitate the growth of other microbes on the electrode surface.

Several studies have shown that sulfide can be oxidized and ultimately deposited on the anode as element sulfur, producing current (Cooney et al., 1996; Rabaey et al., 2006; Dutta et al., 2009; Gong et al., 2013). Here, using differential pulse voltammetry (DPV) for the first time, we provide direct evidence for the oxidation of sulfide, and its role as an electron carrier. Mixed cultures obtained from the marine sediments were inoculated into an electrochemical cell and examined via chronoamperometry. In addition, microbial community analysis was used to examine the impact of sulfide removal on microbial-community composition.

## Materials and methods

### Characteristics of sediments and the mixed cultures

Two sediments collected from Catalina Harbor, Santa Catalina Island (33.4285°N, 118.5090°W) and the Port of Los Angeles (33.7370°N, 118.2703°W) were used in this study. All the sediments were sieved by using a 420-μm sieve to remove plankton, invertebrates, and gravel, as suggested elsewhere (Nielsen et al., 2010). Approximately 70 milligrams of the sieved sediments were added to 30 mL of modified Postgate B medium (Postgate, 1979), incubated anaerobically in serum bottles, and subsequently inoculated into electrochemical cells for further tests. The two mixed cultures from these sediment enrichments were called Catalina-Harbor (CAT) and the Port-of-Los-Angeles (LA), respectively. The organic compositions of the two different sediments were determined by the ignition method (Clesceri et al., 1989), and were 11 and 82 mg g^−1^ for CAT and the LA sediments, respectively. The modified Postgate-B medium contained: phosphate buffer, 3.6 mM; NH_4_Cl, 18.7 mM; MgSO_4_, 16.6 mM; CaCl_2_, 450 μM; sodium lactate, 26 mM; ascorbic acid, 0.6 mM; thioglycolic acid, 1 mM; yeast extract, 1 g L^−1^; artificial seawater powder, 32 g L^−1^; FeSO_4_, 1.7 mM, C_6_H_9_NO_3_, 78.5 μM; MnSO_4_, 29.6 μM; CoCl_2_, 4.2 μM; ZnCl_2_, 9.5 μM;CuSO_4_, 0.4 μM; AlK(SO_4_)_2_, 0.2 μM; H_3_BO_3_, 1.6 μM;Na_2_MoO_4_, μM;NiCl_2_, 1.0 μM;Na_2_WO_4_, 0.8 μM. When the medium was used as an electrolyte in the electrochemical cells, the ingredients of yeast extract, ascorbic acid, and thioglycolic acid were removed, to avoid any additional electrochemical reaction on the electrode surfaces (Marsili et al., 2008; Masuda et al., 2010). In addition, the concentration of FeSO_4_ was reduced to 3.6 μM, to remove the function of impairing sulfide concentration but leave a trace amount for microbial growth. All the enrichments (including serum-bottle and electrochemical enrichements) were conducted at room temperature with initial pH = 7.8.

### Graphite electrodes, electrochemical cell, and chronoamperometric measurement

In the present study, the carbon graphite electrodes (Electrolytica Inc., Amherst, NY, United States) were pretreated by soaking with a 90% ethanol followed by an acid wash with 1 M hydrochloric acid, and dried at 105°C. At each step, the electrodes were rinsed and washed eight times with distilled water within a sonicator, to remove the ethanol and hydrochloric acid.

An asymmetric design of electrochemical cell was used (Supplementary Figure 1), which utilizes 200-mL working-electrode (anode) and 30-mL counter-electrode (cathode) chambers separated by 10 cm^2^ of a proton exchange membrane (Nafion N115, Dupont, Wilmington, DE, United States). The headspaces of the anode and cathode chambers were both continuously flushed with nitrogen gas with a flow rate of 20 mL min^−1^, to maintain anaerobic conditions. A metal mold with Teflon spacers was used to clamp the interface between two chambers and maintain the whole cell configuration. Each cylindrical anode chamber was configured with a cylinder inner diameter of 4.6 cm and a height of 17 cm, and the inner walls was lined with 78 cm^2^ (12.8 cm × 6.1 cm) of pretreated carbon felts fixed with a Teflon spacers. A fine platinum wire was attached outside the carbon felt as a current collector, and an Ag|AgCl|KCl (sat.) reference electrode was inserted in proximity to the carbon felt. The anode chambers were capped tightly with silicon rubber stoppers, and the liquid was mixed with a magnetic stirrer. All items except for the reference electrodes of the reactors were autoclaved at 121°C for 20 min after assembling. The whole device was connected to a potentiostat (Quadstat EA 164, eDAQ Pty. Ltd., NSW., Australia) equipped with a data acquisition system (e-corder, eDAQ Pty. Ltd., NSW., Australia), and the working electrode potential was controlled at +130 and +630 mV (i.e., −100 and +400 mV vs. Ag/AgCl, depending on the experimental conditions). All potentials in this paper are referred to the standard hydrogen electrode. The anode electrolyte consisted of the recipe described in Section Characteristics of Sediments and the Mixed Cultures; the cathode electrolyte consisted of 100 mM of phosphate buffer (pH = 7.8) and 80 mM of KCl.

### Sulfide oxidation examined by differential pulse voltammetry (DPV), cyclic voltammetry (CV), and choroamperometry

A 30-cm^3^ glass cell connected an electrochemical analyzer (Wavenano, PINE, Grove City, PA, United States) was used to study electrochemical sulfide oxidation with the concentrations of 1.6 mM in each tests. In DPV tests, the glassy carbon (GCE, with a diameter of 4.0 mm, CH Instruments, Inc., USA), platinum wire (0.5 mm thick, 5 cm long), and Ag|AgCl|KCl (sat.) electrodes were used as the working, counter, and reference electrodes, respectively (Supplementary Figure 2A). DPV was performed using 5.0-mV pulse increments, 50-mV pulse amplitude, 300-ms pulse width, and a 5.0-s pulse period. Prior to implementing DPV, the GCE would was polished to a mirror-like finish with fine emery papers. In CV (with 10 mV s^−1^ of scanning rate) and chronoamperometry the graphite block (1 cm × 2 cm × 0.6 cm) connected with a platinum wire (0.1 cm thick) was used as a working electrode instead (Supplementary Figure 2B). To achieve different pHs, McIlvaine buffer with the amendment of 80 mM of KCl was used. The concentration of thiosulfate after chronoamperometric tests were analyzed by using a cyanolysis and spectrophotometric method (Kelly et al., 1969). All the tests were run under room temperatures.

### Microbial community analyses

The deoxyribonucleic acid (DNA) was extracted using the PowerSoil DNA kit (MO BIO Laboratories, Inc., Carlsbad, CA, United States) according to the manufacturer's instructions. After each batch test, the biofilm-attaching carbon felts were cut into small pieces with an area of ca. 0.5 × 3.0 cm^2^, and ca. 0.5 g of carbon felt was stuffed into each bead tube. To obtain an adequate DNA concentration, saline dissolved DNA extracted from five bead tubes was concentrated in one 2-mL collection tube with a spin filter, and the final DNA concentrations ranged from 20 to 70 μg μL^−1^. The 16S rRNA gene V4 variable region PCR primers 515/806 with a barcode on the forward primer were used in a 30 cycle PCR using the HotStarTaq Plus Master Mix Kit (Qiagen, Valencia, CA, United States) under the following conditions: 94°C for 3 min, followed by 28 cycles of 94°C for 30 s, 53°C for 40 s and 72°C for 1 min, after which a final elongation step was performed at 72°C for 5 min. Multiple samples were pooled together in equal proportions based on their molecular weights and DNA concentrations. Pooled samples were purified using calibrated Ampure XP beads (Beckman Coulter Inc., Brea, CA, United States). The pooled and purified PCR product was then used to prepare a DNA library by following the Illumina TruSeq DNA library preparation protocol. Sequencing was performed at MR DNA (www.mrdnalab.com, Shallowater, TX, USA) on a MiSeq following the manufacturer's guidelines. Sequence data were processed using the Molecular Research DNA Laboratory analysis pipeline (Shallowater, TX, United States). Operational taxonomic units (OTUs) were defined by clustering at 3% divergence (97% similarity). Final OTUs were taxonomically classified using BLASTn against a curated database derived from GreenGenes, RDPII and NCBI [www.ncbi.nlm.nih.gov, DeSantis et al. (2006), http://rdp.cme.msu.edu].

### Specimens pretreatment and scanning electronic microscope (SEM) observation

The specimens (i.e., biopellets attached on graphite felt) were fixed in a solution of 2.5% glutaraldehyde and PBS (pH 7.8) for 16 h at 4°C. Specimens were rinsed three times in the PBS to remove residual glutaraldehyde, for 10 min each time. Dehydration was carried out with a series of gradually increasing ethanol concentrations: 50, 75, 85, 95, and 100%. Finally, the dehydrated specimens were dried using the critical point drier (Tousimis 815, Rockville, MD, United States) and then coated with gold by an ion sputter (JEOL JFC-1100, JEOL Ltd., Tokyo, Japan). Desiccated samples were observed by using a JEOL JSM-7001 SEM (JEOL Ltd., Tokyo, Japan).

### Instrumental analyses

Organic acids (i.e., lactate and acetate) contained in the cultural medium were detected using high-performance liquid chromatography (1100 Series, Agilent Technologies Inc., Santa Clara, CA, United States) equipped with a quaternary pump, a UV-visible photo diode array detector, and a reversed-phase column (Synergi™ 4 μm Hydro-RP 80 Å, Phenomenex, Torrance, CA, United States). 4.5 mM sulfuric acid solution was used as a mobile phase at a flow rate of 1 mL min^−1^. The organic acids were detected at a specific wavelength of 210 nm. Concentrations of sulfate were determined using an ion chromatograph (Metrohm 850 Professional IC Anion, Herisau, Switzerland) equipped with an anion Metrosep A Supp 5—250/4.0 column operated under 40°C. A solution containing 3.2 mM of Na_2_CO_3_, 1 mM of NaHCO_3_, and 2.5% of acetonitrile was used as the eluent at a flow rate of 0.8 mL min^−1^. Two point five % of acetonitrile was used as the regenerant which is used to regenerate the resins packed in the suppressor.

## Results

### Electrochemical characteristics of sulfide oxidation on the glassy carbon electrodes

**Figure 1:**
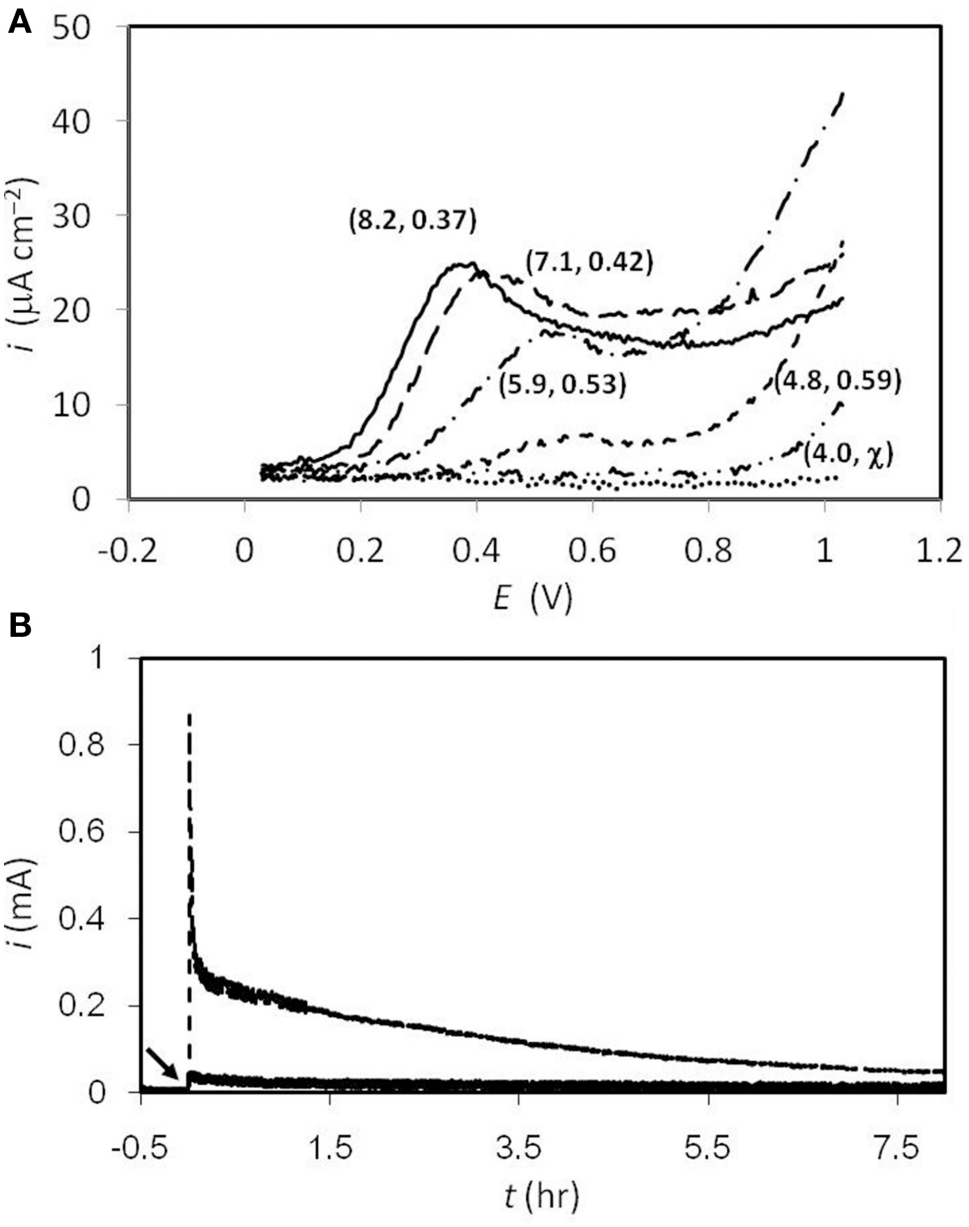
**(A)** Differential pulse voltammogram of sulfide oxidation at different pHs. The numbers in the parentheses denote (actual pH value, peak potential). Solid line, pH 8; dash line, pH 7; dash-dot line pH 6; short dash line, pH 5; dash-dot-dot line, pH 4; dot line, sulfide-free test at pH 8. **(B)** Responding current during electrochemical sulfide oxidation with 630 mV poised on the graphite electrodes. The broken and solid lines represent the results collected at pH = 8 and 4, respectively. The arrow indicates high concentration of sulfide spiked (resulting 1.6 mM) at *t* = 0 hr.

Glassy carbon was used in the present study to investigate sulfide oxidation, which has been widely used in electrochemical experiments due to its uniform carbon structure (Brett and Brett, 1993). All the tests at different pHs were carried out by adding 1.6 mM of sulfide, while the blank control was done in McIlvaine buffer (pH = 8) with no sulfide addition. As shown in Figure 1A, at pH = 8, a sulfide-oxidizing current was observed at +30 mV, while the peak current goes to 25 μA cm^−2^ at the potential of +0.37 V. From pH = 8 to 5, the potentials where the peak current could be observed were +370, +420, +530, and +590 mV, respectively. The peak currents decreased when the pH decreased, while no peak current was seen at pH = 4. With regard to the result obtained at pH = 8, when the potential was scanned to higher than 900 mV, the response current kept going up, instead of decaying to the background level. Similar trends were found at all pH conditions. The current of the primary peak decreased when the pH level went lower, and no obvious primary peak was observed at pH = 4. On the other hand, the “tailing” current continued increasing at lower pHs, indicating the occurrence of an additional reaction, and this was correlated closely to the amount of non-ionized sulfide. The continuous sulfide oxidation with 630 mV poised was also tested abiotically, as the results exhibited in Figure 1B. After 8 h of reaction, 4.2 and 0.6 Coulombs of charge were collected at pH = 8 and 4, respectively, and samples were also taken at the end of the tests for thiosulfate analyses. However, the concentrations of the thiosulfate were all beneath the detectable level (<0.1 mM) under both conditions.

### Current generation of sedimentary mixed culture with different poised potentials

**Figure 2:**
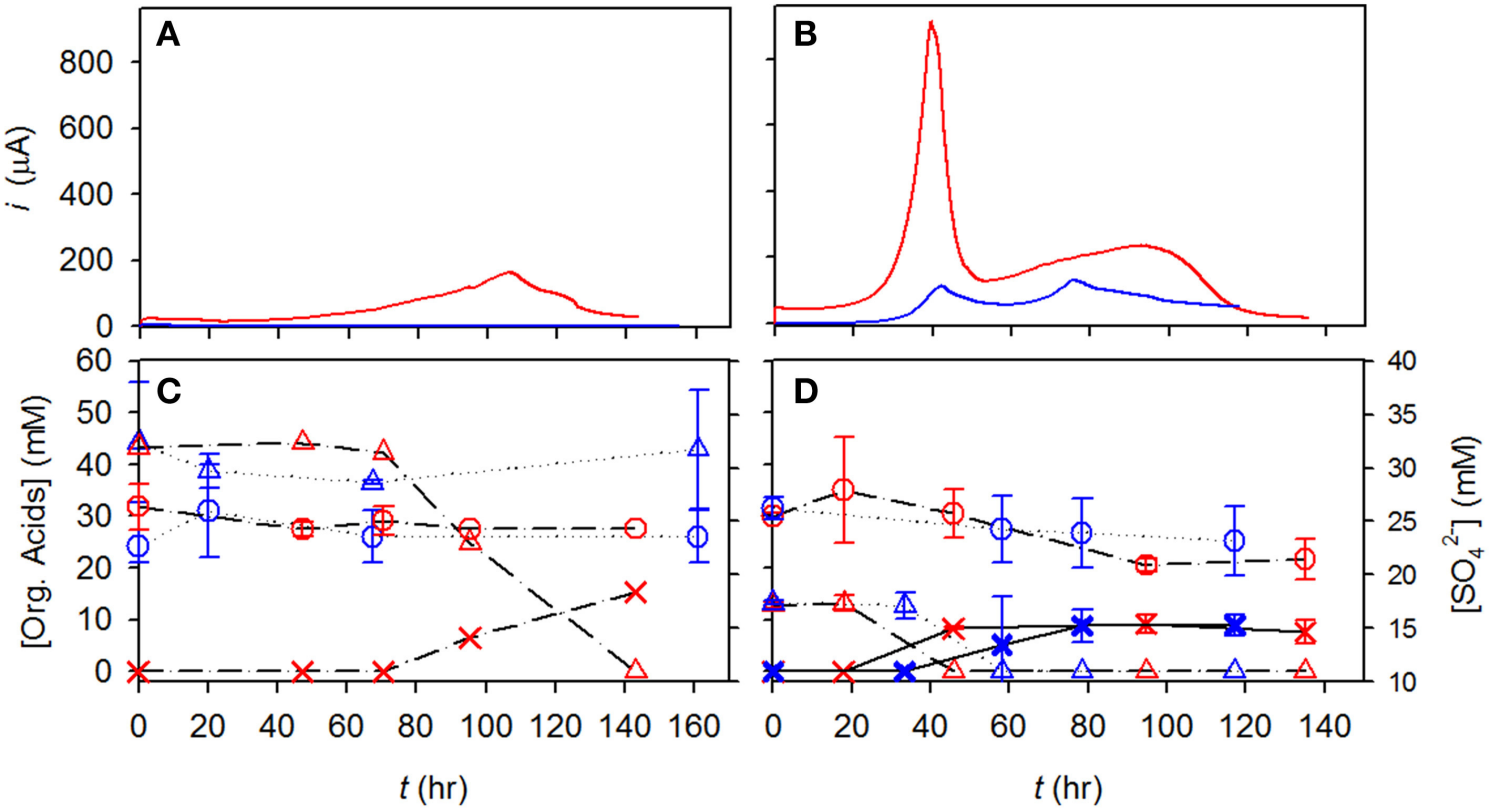
**Time course profiles of current, lactate, and acetate for CAT and LA tested in electrochemical cells**. **(A,B)** Current obtained at +630 mV, red line; current obtained at +130 mV, blue line; **(C,D)** lactate at +630 mV, red triangle; sulfate at +630 mV, red circle; acetate at +630 mV, red X; lactate at +130 mV, blue triangle; sulfate at +130 mV, blue circle; acetate at +130 mV, blue X.

As described in the Materials and Methods section, both CAT and LA sediments were used to examine the effect of electrochemical sulfide oxidation on changes in the microbial community (Figure 2). Different sizes of graphite felts, different concentrations of lactate, and different amounts of inoculation were used in these batch tests (i.e., 6 mm thick of graphite, 40 mM of lactate, and 34 mg L^−1^ of inoculated volatile solids were used for the CAT test; 3 mm thick of graphite, 12 mM of lactate, and 12 mg L^−1^ of inoculated volatile solids were used for the LA test, respectively), to deal with the limitations of current reading due to the different levels of bio-activities. After ca. 140 h of incubation in the CAT test, neither lactate nor sulfate was significantly converted. At a potential of +630 mV, lactate was not degraded until 70 h, and subsequently decreased to zero at 140 h, producing 20 mM of acetate. During this time, only 1 mM of sulfate was consumed. In the LA tests, the degradation rates of lactate were 0.62 and 0.58 mM h^−1^, with electrodes poised at +630 and +30 mV, respectively. The final concentrations of acetate was 10 mM in both conditions, while the sulfate concentration decreased by only 4 mM, from 25 to 21 mM.

### Microbial community analyses and SEM observation

**Figure 3:**
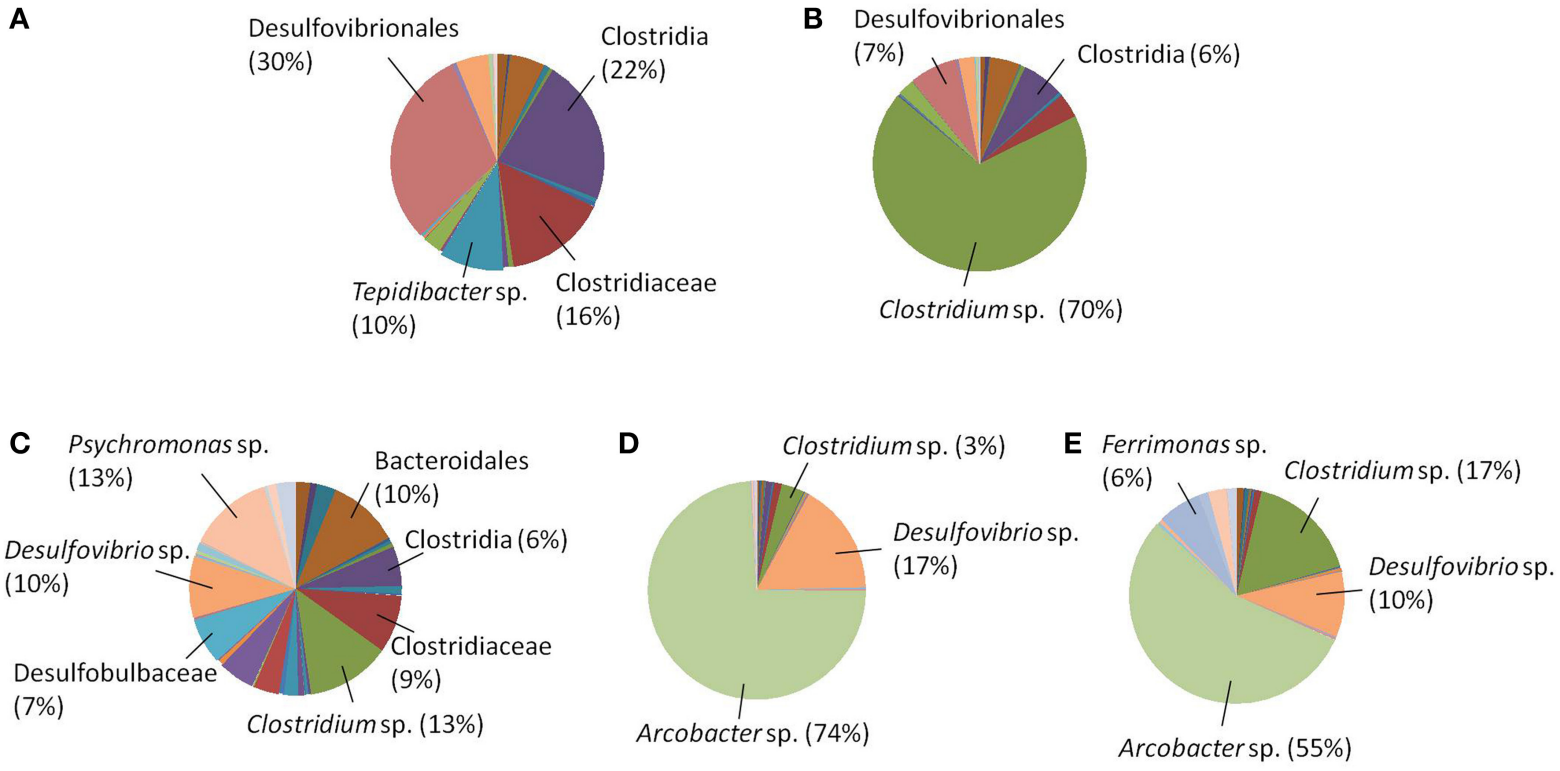
**Microbial community analyses (A) CAT in serum bottles; (B) CAT in electrochemical cells, +630 mV; (C) LA in serum bottles; (D) LA in electrochemical cells, +130 mV; (E)LA in electrochemical cells, +630 mV**.

**Figure 4:**
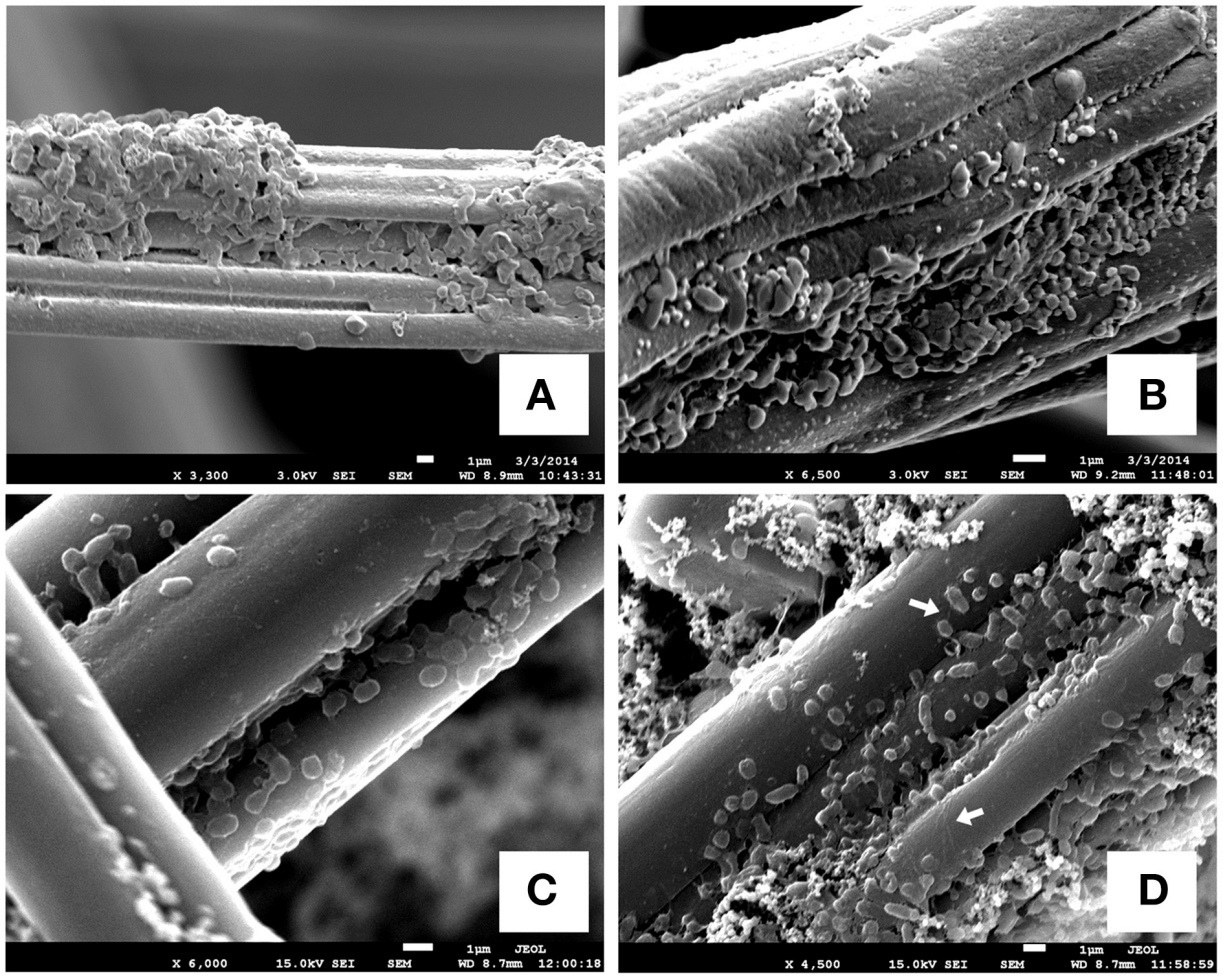
**SEM images of biofilms on graphite-felt electrodes**. **(A)** Developed biofilms of CAT under the condition of +630 mV; **(B)** developed biofilms of LA under the condition of +130 mV; **(C,D)** developed biofilms of LA under the condition of +630 mV that some nano-wire-like structures could be seen. The white arrows indicate the significant nanowire appendages lining on graphite fibers.

In addition to the reaction profiles during electrochemical incubation, microbial community analyses were carried out to compare the microbial population composition before and after electrochemical incubation. The results shown in Figures 3A,C represent the microbial communities of CAT and LA obtained after enrichment by using yeast extract containing media. Figures 3B,D,E are the results for CAT and LA enriched in the electrochemical cells with +130 and +630 mV applied to each condition. No microbial community analysis was done for CAT, as no significant reactions were found with +130 mV poised. In the serum bottle cultivated CAT, the microbial community was dominated by bacteria affiliated with *Desulfovibrionales* (30%), followed by *Clostridia* (22%), *Tepidibacter* sp. (10%), and Clostridiaceae (16%); The LA-containing serum bottles were dominated by *Psychromonas* sp. (13%) and *Clostridium* sp. (13%), followed by *Desulfovibrio* sp. (10%), *Bacteroidales* (10%), Clostridiaceae (9%), Desulfobulbaceae (7%), and Clostridia (6%). The samples for electrochemical enrichment were taken after 140 h of incubation. The CAT samples maintained at +630 mV were dominated by *Clostridium* sp. (70%), followed by *Desulfovibrionales* (7%) and *Clostridia* (6%). In contrast, the LA samples maintained at 130 mV or +630 mV potentials were dominated by *Arcobacter* sp., at 74 and 55%, respectively. From +130 to +630 mV, the composition of *Clostridium* sp. was only 3% at +130 mV, and this increased to 17% at +630 mV, whereas the composition of *Desulfovibrio* sp. decreased from 17 to 10% from +130 to +630 mV. In addition, bacteria affiliated to *Ferrimonas* sp. were seen only under the +630 mV condition. To investigate the morphology of the mixed cultures grown on carbon felt, SEM observations were conducted after 180 h of electrochemical culturing (Figure 4). The SEM micrographs in Figures 4A,B show the microorganisms growing on graphite fibers with versatile morphologies, indicating that two mixed cultures were successfully cultivated on the electrode surfaces. Figures 4C,D show the nanowire-like appendages when enriching LA under the condition of +630 mV, and this has been reported as a special microbial morphology that may possess many c-type cytochromes that can be used to facilitate EET (Gorby et al., 2006; Pirbadian et al., 2014). Besides, the sulfur crystal structure caused by sulfide oxidation that was found in Dutta et al. (2009) was not observed in the present study.

## Discussion

The heterotrophic microorganisms in sediments are strongly coupled to the redox processes involved with the mineralization of organics and the reduction of electron acceptors, thereby playing important roles in the global carbon cycle (Kan et al., 2011). Electrodes with poised potentials can thus be used to quantify the redox equivalents delivered to solid-state electron acceptors in the form of current. EET-capable bacteria can interact with poised electrodes, with increased swimming speeds at higher potentials (Harris et al., 2010, 2012), methods of accumulation around these electrodes, and cell growth and biofilm formation (Carmona-Martinez et al., 2013). These phenomena are all related to the interactions between bacterial cytochromes and electrode potentials, as evidenced by testing specific-gene-deletion mutants and CV. It is, thus not surprising that *Ferrimonas* sp., which has been reported to be closely related to *Shewanella* species and capable of metal respiration (Rossellomora et al., 1995; Nakagawa et al., 2006), increased in abundance upon incubation with a +630 mV electrode. In addition, we also suggest that *Ferrimonas* sp. might be the possible candidate to produce nanowire as shown in Figure 4, due to its capability if metal respiration. Many reports of microbial communities of heterotrophs on bio-anodes have concluded that anodes will enrich EET-capable bacteria (Torres et al., 2009; Futamata et al., 2013; Ketep et al., 2013; Babauta et al., 2014; Croese et al., 2014; Miceli et al., 2014; Zhu et al., 2014). Nevertheless, because these studies used well-defined media as electrolytes (i.e., with the buffers, substrates, conductivities, and the other growth factors at the necessary levels), the results cannot reveal what happens in practice in natural environments. Especially, for example, the composition of seawater is extremely complicated, and thus some additional reactions might biotically or abiotically occur on the electrodes [e.g., element sulfur deposition (Dutta et al., 2009), and iron-sulfide might form, Nakamura et al. (2010)], thus creating some other electron transfer mechanisms instead of, or in addition to, the cytochrome-mediated ones. Therefore, we suggest that prior to any *in situ* applications (Friedman et al., 2012), the surroundings of the electrodes should be thoroughly investigated to understand the contributions of background abiotic currents. Exactly how this should be done needs to be worked out, as experiments with sediment killed controls are difficult.

Based on our preliminary work (Supplementary Figure 3), CV tests were carried out to analyze the sulfide oxidation on graphite electrodes. According to the results, a significant oxidizing current started at an onset of +30 mV. Concurrently, the peak current of ca. 1 mA was found at +430 mV. The peak current decreased when increasing the CV cycles, indicating that while the sulfide readily oxidized there was no corresponding abiotic (reversible) reduction reaction within the scanning interval. This result agrees very well with a previous study (Liang et al., 2013). In addition to CV, DPV provides an efficient way to impair the non-Faradic charging current on the electrode surface, which renders the voltammogram more reliable and informative. In particular, if a small surface area (ca. 0.1 cm^2^) electrode is used, then this can minimize interference from the non-Faradic charging current, and thus produce more accurate results. According to the results shown in Figure 1A, at all pHs the currents did not go back to the background levels as revealed in the sulfide-free test (i.e., the dotted line in Figure 1A), which implies that the sulfide could be converted to other oxidized species, rather than only to element sulfur (e.g., thiosulfate). The potential of the peak current to shift along with the change in pH is expected, as sulfide oxidation is associated with proton release. However, the DPV and chronoamperometry results (Figures 1A,B) indicate that the efficiency of oxidizing sulfide became worse at low pH. This might be due to the hydrophobic characteristic of the non-ionized sulfide that forms at low pH, which might inhibit sulfide oxidation on the electrode surface. With regard to thiosulfate analyses, the results of low amount converted (<1 mM) are similar to the results reported by Dutta et al. (2009). In addition, the results in Figure 1B exhibit that even with a high potential poised (i.e., 630 mV), 1.6 mM of sulfide was not oxidized readily and required more than 8 h to be depleted. It is suggested that the sulfur chemistry on polarized electrodes could be further studied, for the sake of clarifying the mechanisms and enhancing sulfide-removal rate on anodes.

The presence of metals (e.g., iron) in culture medium is usually required to remove the sulfide produced by SRB (Widdel, 1983; Madigan et al., 2012). In addition, the low level of sulfide (ca. 0.6–3 mM) generated by SRBs also inhibits other microorganisms with regard to developing consortia [e.g., anaerobic digestor, Speece (1983) and Parkin et al. (1990), and marine bacteria (Mirzoyan and Schreier, 2014)], which can present problems when trying to study microbial consortia including SRBs. However, some SRBs can tolerate sulfide concentrations of up to 16 mM (Reis et al., 1992) which is higher than the previously reported level. In the present study, sulfide oxidation is similar to removing inhibitors for the other microorganisms that are not SRBs. Therefore, the abundance of SRB in the CAT or LA cultures did not significantly increase after electrochemical enrichment (i.e., from 30 to 7% for *Desulfovibrionales* in CAT; from 10 to 17% or 10% for *Desulfovibrio* sp. in LA). It is noteworthy that, the amount of volatile solids in the sediment of Port of Los Angeles is much greater than that in the sediment of Catalina Harbor (shown in Section Characteristics of Sediments and the Mixed Cultures). This implies that a higher level of heterotrophic-sulfate-reducing activity might be driven by the organic-rich environment in the sediment of the Port of Los Angeles, resulting in a considerable amount of sulfide generated *in situ*, and enabling the indigenous microorganisms to tolerate higher levels of this. This could explain why the tendency of lactate degradation does not change significantly when enriching LA electrochemically, no matter whether +30 or +630 mV of potentials is applied.

According to the results obtained after the electrochemical enrichment, some bacteria which are not EET-capable, like *Clostridium* sp. and *Arcobacter* sp., became dominant in CAT and LA, respectively. *Clostridium* species have been reported as the dominant populations on polarized graphite electrodes in many studies (Rismani-Yazdi et al., 2007; Timmers et al., 2012; Zhang et al., 2012). However, the discussion of the their EET capabilities was not sufficiently given, but some results with regard to using coating catalysts [i. e., poly(tetrafluoroaniline), which is used to convert hydrogen produced by *Clostridium* sp., (Niessen et al., 2004)] were quoted incorrectly. In addition, the *Arcobacter* species has only been reported as an electrochemically active bacterium in a few other studies (Fedorovich et al., 2009; Pereira-Medrano et al., 2013). While the factors leading to the enrichment of any given group cannot be specified at this time, the potential of the electrode surface provides some advantages to these microbes, making them more likely to be associated with the consortia that are already there.

Electrochemical sulfide oxidation was used in the present study to remove sulfide continuously, and this process can be used to avoid the addition of confined metal when removing sulfide during continuous culture. Unlike traditional methods, because of the accumulation of sulfide, enrichments for microorganisms commonly involve a series of transfers to sulfide-free media. In contrast, using the approach proposed in this work, and continually removing sulfide, may make it possible to find and cultivate previously uncultivated microbial strains for physiological study. We believe that the use of this electrochemical approach will lead to a better understanding of how and why microorganisms exchange electrons with solid surfaces, and how this ability can be used for the cultivation, understanding, and exploitation of microbes from many different environments.

### Conflict of interest statement

The authors declare that the research was conducted in the absence of any commercial or financial relationships that could be construed as a potential conflict of interest.
