# Supplementary material for: Enriching distinctive microbial communities from marine sediments via an electrochemical-sulfide-oxidizing process on carbon electrodes
Source: Front Microbiol. 2015 Feb 17;6:111. doi: 10.3389/fmicb.2015.00111 (PMC4330880; doi:10.3389/fmicb.2015.00111)
Supplement: Supplementary file 1 [file DataSheet1.DOCX]

***Supplementary Material***

**Enriching distinctive microbial communities from marine sediments via an electrochemical-sulfide-oxidizing process on carbon electrodes**

**Shiue-Lin Li^1^, Kenneth H. Nealson^1, *^**

^1^Department of Earth Science, University of Southern California, Los Angeles, CA, USA

*** Correspondence:** Dr. Kenneth H. Nealson, University of Southern California, Department of Earth Science, 835 Bloom Walk, SHS562, Los Angeles,CA90089, USA

knealson@usc.edu

**Supplementary Figures and Tables**


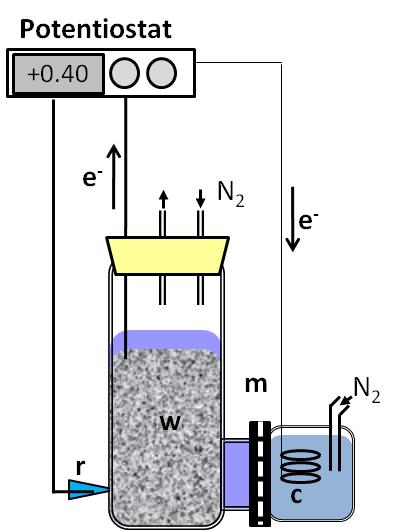


Supplementary Figure 1. Schematic diagram of an bioelectrochemical cell. w, carbon felt working electrode; c, Pt wire counter electrode; r, Ag|AgCl|KCl (saturated) reference electrode; m, Nafion N115 membrane.


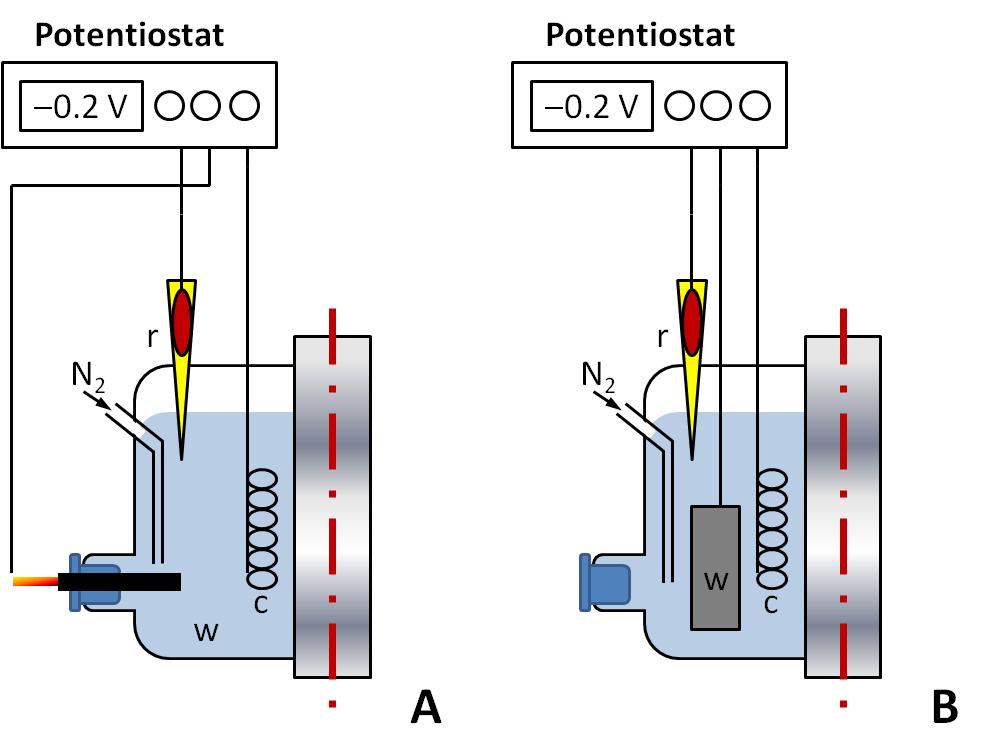


**Supplementary Figure 2.** Schematic diagram of an bioelectrochemical cell used for (A) DPV and (B) CV measurements. w, glassy-carbon/graphite working electrode; c, Pt wire counter electrode; r, Ag|AgCl|KCl (saturated) reference electrode.

**Supplementary Figure 3.** Cyclic voltammograms in deoxygenated phosphate buffers. The red curve was carried out with of 1.6 mM of Na_2_S in a quiet state. The scanning rate was 10 mV s^−1^.
